# Pleiotropic neurotransmitters: neurotransmitter-receptor crosstalk regulates excitation-inhibition balance in social brain functions and pathologies

**DOI:** 10.3389/fnins.2025.1552145

**Published:** 2025-03-14

**Authors:** Anping Chai

**Affiliations:** Shenzhen Key Laboratory of Translational Research for Brain Diseases, The Brain Cognition and Brain Disease Institute, Shenzhen Institute of Advanced Technology, Chinese Academy of Sciences, Shenzhen-Hong Kong Institute of Brain Science-Shenzhen Fundamental Research Institutions, Shenzhen, Guangdong, China

**Keywords:** neurotransmitter, synaptic transmission, excitation-inhibition balance, autism spectrum disorder, schizophrenia, epilepsy

## Abstract

Neuronal excitation-inhibition (E/I) balance is essential for maintaining neuronal stability and proper brain functioning. Disruptions in this balance are implicated in various neurological disorders, including autism spectrum disorder, schizophrenia and epilepsy. The E/I balance is thought to be primarily mediated by intrinsic excitability, governed by an array of voltage-gated ion channels, and extrinsic excitability, maintained through a counterbalance between excitatory synaptic transmission primarily mediated by excitatory transmitter glutamate acting on excitatory ion-tropic glutamate receptors and inhibitory synaptic transmissions chiefly mediated by GABA or glycine acting on their respective inhibitory ion-tropic receptors. However, recent studies reveal that neurotransmitters can exhibit interactions that extend beyond their traditional targets, leading to a phenomenon called neurotransmitter-receptor crosstalk. Examples of such crosstalks include earlier discovery of inhibitory glycine functioning as co-transmitter gating on the NMDA subtype of excitatory glutamate receptor, and the most recent demonstration that shows the excitatory glutamate transmitter binds to the inhibitory GABAA receptor, thereby allosterically potentiating its inhibitory function. These studies demonstrate structurally and physiologically important crosstalk between excitatory and inhibitory synaptic transmission, blurring the distinction between the concepts of classic excitatory and inhibitory synaptic transmission. In this article, evidence supporting the forms of excitatory and inhibitory crosstalks will be briefly summarized and their underlying mechanisms will be discussed. Furthermore, this review will discuss the implications of these crosstalks in maintaining the E/I balance, as well as their potential involvement in synaptic plasticity and cognition in the context of social conditions.

## 1 Introduction

The brain relies on neurotransmitters as messengers to enable precise and efficient communication between neurons. These neurotransmitters are released and bind to receptors, conveying signals that regulate processes including neural development, synaptic plasticity and excitation-inhibition (E/I) of membrane potentials. Disruptions in the E/I balance are believed to be central to the pathogenesis of various neurological disorders, including schizophrenia and autism spectrum disorder (ASD) characterized by social deficits, as well as epilepsy (Fritschy, 2008; Lee et al., 2017; Liu et al., 2021). The E/I balance is primarily regulated by intrinsic excitability, which is controlled by a range of voltage-gated ion channels, and by extrinsic excitability, which is maintained through a balance between excitatory and inhibitory synaptic transmissions (Desai, 2003; Chen L. et al., 2022). In the mammalian central nervous system, excitatory synaptic transmission is primarily driven by the neurotransmitter glutamate, which acts on excitatory ionotropic glutamate receptors, while inhibitory synaptic transmission is predominantly mediated by *γ*-aminobutyric acid (GABA) or glycine, which act on inhibitory ionotropic GABAA receptor (GABA_A_R) or glycine receptor (GlyR) respectively (Hyman, 2005). Increasing evidence, however, reveals that neurotransmitter could bind to receptors outside their conventional pairings as mentioned before under certain conditions (Liu et al., 2010; Bianchi et al., 2011; Wen et al., 2022; Piot et al., 2023). This phenomenon of neurotransmitter-receptor crosstalk adds another layer of complexity to the regulation of information processing in the brain. It is plausible that neurotransmitter-receptor crosstalk disturbance may also contribute to E/I imbalance and synaptic plasticity deficit. Therefore, a deeper understanding of mechanism of neurotransmitter-receptor crosstalk would inform the development of therapeutic strategies for treating social disorders related to E/I imbalance.

## 2 Neurotransmitter-receptor crosstalk

Neurons communicate primarily via chemical synapses. Typically, the presynaptic neuron releases neurotransmitters, which are stored in vesicles at the axon terminal, transmitting signals to ionotropic or metabotropic receptors on the postsynaptic neuron to facilitate information transfer. This review focus on three types of ionotropic receptors, including the N-methyl D-aspartate receptor (NMDAR) which is cationic receptor, and GABA_A_R, GlyR which are anionic receptors (Table 1). Each ligand listed in the table could target more than one type of ionotropic receptor, and each ionotropic receptor was the target of more than two kinds of ligand. It is well-known that glycine acts both as an agonist on inhibitory GlyR and as a co-agonist of excitatory NMDAR in the spinal cord and brain. Surprisingly, recent studies have demonstrated that glutamate, a canonical excitatory transmitter, could positively modulate inhibitory GABA_A_R function in heterologous expression system and hippocampal slices of mice (Wen et al., 2022). Furthermore, one study reported that glutamate could directly activate GlyR as a positive allosteric modulator in spinal cord neurons (Liu et al., 2010), but the other study did not find the direct link (Aubrey et al., 2020). Additionally, other studies have reported that histamine, a canonical transmitter of histamine receptors, could also potentiate NMDAR in cultured hippocampal neurons or recombinant GABA_A_R in HEK cells (Burban et al., 2010; Bianchi et al., 2011). It should also be noted that neurotransmitter-receptor crosstalk could occur with non-ionotropic receptors. For example, Piot *et al.* revealed that GABA, a canonical inhibitory transmitter, could enhance glutamate delta-1 receptor (GluD1) function and long-term potentiation of inhibitory synaptic plasticity in mouse hippocampus (Piot et al., 2023). Overall, as shown in Table 1, this type of transmitter-receptor crosstalk is therefore not an exception but could be considered as a common phenomenon in the nervous system.

**Table 1:** Neurotransmitter-receptor crosstalk in animal models.

Receptor	Transmitter	Effect	E/I balance	Animal model	Brain diseases
NMDAR (Na^+^, K^+^, Ca2^+^)	Glutamate	Agonist	Excitation↑	*Grin1*^D481N^ mutant mice	Schizophrenia
	Glycine	Co-agonist	Excitation↑	*Grin1*^neo−/−^ mice	ASD
GABA_A_R (Cl^-^)	GABA	Agonist	Inhibition↑	*Gabrb3*^S408/409A^ KI mice	ASD
	Glutamate	Modulator	Inhibition↑	*Gabrb3*^E182G^ or *Gabrb2*^E181G^ KI mice	Epilepsy
GlyR (Cl^-^)	Glycine	Agonist	Inhibition↑	*Glra2* KO mice	ASD
	GABA	Partial agonist	Inhibition↑		
	Glutamate	Modulator	Inhibition↑		

## 3 Molecular mechanisms of neurotransmitter-receptor crosstalk

The molecular mechanisms of glycine acting as co-agonist of NMDAR have been summarized in other reviews (Hanson et al., 2024). Mutations in GluN1, such as D481N, significantly reduce glycine binding affinity in recombinant GluN1/GluN2A receptor and homozygous *Grin1*^D481N^ transgenic mice (Wafford et al., 1995; Kew et al., 2000). Here, the review will focus on glutamate-GABA_A_R and GABA/glutamate-GlyR.

### 3.1 How does glutamate modulate GABA_A_R?

The GABA_A_R is a heteropentameric ionotropic receptor primarily responsible for mediating Cl^−^ /HCO_3_^−^ current in the brain. The heteropentamer is composed of a combination of 19 homologous subunits from eight classes (α1–6, β1–3, γ1–3, δ, ε, θ, π and ρ1–3). Most common configuration consisting of two α and three β aligned β-α-β-β-α, or two α, two β, and one γ subunits aligned β-α-γ-β-α counter-clockwise when viewed from the extracellular side. The GABA binding sites are located at the interface between the α and β subunits, specifically at the β+/α- interface (Sieghart and Savic, 2018).

Over 30 years ago, it was discovered that the excitatory neurotransmitter glutamate potentiated GABA_A_R current in acutely isolated hippocampal neurons (Stelzer and Wong, 1989). A recent study identified a novel mechanism through which glutamate can directly modulate the inhibitory GABA_A_R via allosteric potentiation (Wen et al., 2022). By combining electrophysiological recordings, radioligand binding assays, molecular modeling and mutational analysis, Wen et al. demonstrated that glutamate directly potentiated GABA-evoked currents in recombinant α1β2 GABA_A_R, with an EC_50_ of approximately 180 μM, comparable to nearly 1 mM concentration of glutamate in the synaptic cleft during neurotransmission (Clements et al., 1992). It is noteworthy that this potentiation was also mimicked by glutamate analogs, such as AMPA, kainate, and NMDA, as well as by the NMDA receptor antagonist AP5 in recombinant *α*1β2 and α1β2γ2 GABA_A_R, indicating some structural specificity for glutamate-like molecules. Molecular modeling and mutational analysis identified a novel glutamate binding pocket at the α+/β- subunit interface. Key residues involved in forming this pocket included K104, E137, and K155 on the α1 or α2 subunit, E181 on the β2 subunit and E182 on the β3 subunit. The mutations of these sites largely eliminated glutamate potentiation without affecting GABA binding affinity on GABA_A_R.

### 3.2 How does GABA or glutamate modulate GlyR?

GlyR is a pentameric receptor that is also permeable to chloride ions. In mammals, there are four α subunit isoforms (α1-α4) and one β subunit. Functional GlyRs can be either homomeric α or heteromeric αβ composition. The EC_50_ for glycine on recombinant GlyRs generally ranges from 25 μM to 280 μM for α1 and from 46 μM to 541 μM for α2, a typical value for heteromeric receptors is around 100 μM (De Saint Jan et al., 2001; Legendre et al., 2009), which is significantly higher than the glycine concentration at NMDAR. The glycine binding sites are located at the interfaces between the N-terminal domains of neighboring subunits.

De Saint Jan et al. initially reported that GABA could potentiate recombinant human α1 and α2 homomeric glycine receptors current with an EC_50_ of 14.4–160 mM and 64–200 mM, respectively (De Saint Jan et al., 2001). More recently, GABA was identified as a weak partial agonist of GlyR in neurons from the medial nucleus of the trapezoid body (MNTB) of rats aged P10-P18 (Lu et al., 2008). Electrophysiological recordings, conducted in the presence of GABA receptors and glutamate receptors inhibitors or Zn^2+^ chelator, revealed that GABA significantly accelerated the decay kinetics of glycine-evoked currents. In MNTB patches, co-application of 10 mM GABA with 1 mM glycine (at physiological concentration) shortened the fast decay time constant and reduced the peak amplitude of glycine-evoked currents by about 25%. Although GABA may overlap with glycine binding sites, the exact binding sites on GlyR remain unclear.

Regarding the interaction between glutamate and GlyR, Liu et al. provided compelling evidence for a novel allosteric potentiation of GlyR-mediated chloride currents by glutamate [Liu et al., 2010; but also see Borghese et al. (2012); Aubrey et al. (2020)]. The glutamate binding sites on GlyR need to be determined.

All the cases of neurotransmitter-receptor crosstalk mentioned above suggested that neurotransmitters often act synergistically to enhance receptor function. This cooperative action of multiple neurotransmitters on a single receptor type indicates that neural signaling is highly cooperative. Therefore, modulating the non-canonical interactions (e.g., glutamate enhancing GABA_A_R) could provide new avenues for drug development that fine-tune synaptic activity without directly overstimulating canonical pathways. Investigating the non-canonical binding sites and developing compounds that target these sites will expand the toolbox for studying the physiological and pathological engagement of neurotransmitter-receptor crosstalk in the future.

## 4 Where and when does neurotransmitter-receptor crosstalk happen?

Under physiological condition, excitatory and inhibitory nerve terminals are traditionally believed to be strictly segregated to ensure precise function of neural networks and E/I balance. However, accumulating evidence suggested that the E/I balance could also be modulated by mechanisms of heterosynaptic interplay and neurotransmitter-receptor crosstalk. Heterosynaptic interplay, particularly local dendritic excitatory and inhibitory synaptic crosstalk, has been documented both *in vitro* and *in vivo* (Chen et al., 2012; Hayama et al., 2013; Field et al., 2020; Ravasenga et al., 2022). The role of glutamatergic and GABAergic interplay in modulating plasticity and E/I balance has been discussed in several excellent reviews (Chapman et al., 2022; Cupolillo et al., 2024). Such synaptic interplay highlights the synaptic crosstalk occurrence between adjacent synapses within a 3–10 μm space. As for neurotransmitter-receptor crosstalk, it is unlikely that glutamate released from glutamatergic synapse would diffuse and potentiate inhibitory postsynaptic GlyR or GABA_A_R, given the submillimolar/millimolar concentration required for most neurotransmitter-receptor crosstalks. Here, recent findings suggested two conditions may facilitate this crosstalk.

### 4.1 Co-releasing synapse

Neurotransmitters are synthesized by biosynthetic enzymes in neurons. For example, glutamic acid decarboxylase (GAD) catalyzes the conversion of glutamate to GABA. After synthesis, neurotransmitters are transported and concentrated to reach millimolar level in synaptic vesicles (SVs; Edwards, 2007). The uptake of neurotransmitters into SVs is governed by specific vesicular transporters (VTs). These include vesicular glutamate transporters VGLUT1, VGLUT2, and VGLUT3 for glutamate, vesicular GABA transporter/vesicular inhibitory amino acid transporter VGAT/VIAAT for GABA and glycine (Blakely and Edwards, 2012). Distinct VTs in the same SVs are essential for corresponding neurotransmitter acting in neurotransmitter-receptor crosstalk. That is, the co-localization of VGLUT and VGAT in the same SV is necessary for glutamate-GABA_A_R crosstalk. Neurotransmitters are then released through a calcium-dependent SV membrane fusion process triggered by presynaptic depolarization. This section focuses on co-expression of VGLUT and VGAT in the same SV, a phenomenon which occurs more frequently in the brain (Upmanyu et al., 2022).

**Figure 1:**
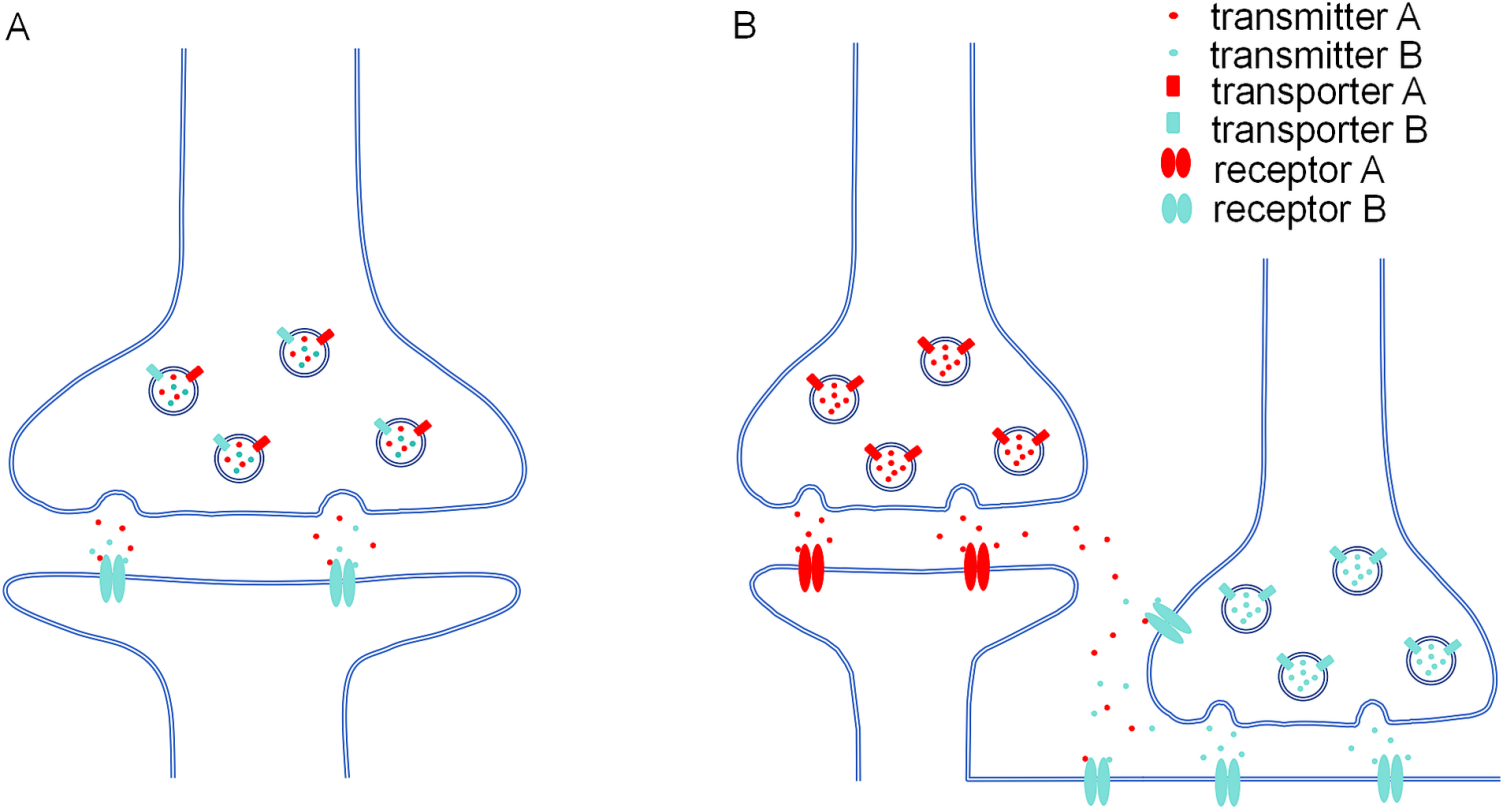
Two possible conditions in which neurotransmitter-receptor crosstalk occurs. **(A)** At the co-release synapses which two types of transmitters (e.g., glutamate and GABA) are co-packaging in the same presynaptic vesicles due to co-localization of two kinds of synaptic vesicular transporter (e.g., VGLUT and VGAT). Postsynaptic receptor binds to both transmitters (e.g., glutamate could allosterically potentiate GABA dependent GABA_A_R current). **(B)** Certain physiological or pathological conditions would cause neurotransmitter spillover, both transmitter A and transmitter B would bind to extrasynaptic or presynaptic receptor B (e.g., high frequency activity would induce glutamate and GABA spillover on extrasynaptic or presynaptic GABA_A_R).

Regarding the allosteric potentiation of GABA_A_R by glutamate, accumulating evidence supported GABA/glutamate co-release in subsets of GABAergic synapses (Figure 1A). (1) Using electron microscopic postembedding immunogold, Stensrud et al. revealed co-expression of VGLUT3 and VGAT in the same vesicle membrane of GABAergic terminals in the hippocampus and cortex of rats and mice (Stensrud et al., 2013; Stensrud et al., 2015), indicating GABA/glutamate co-packaging in same vesicle. Following work by Pelkey et al. showed that VGLUT3/VGAT overlapping in cholecystokinin (CCK^+^) expressing hippocampal CA1 and CA3 interneurons was minimal in early postnatal days, but gradually increased from the juvenile stage to adulthood (Pelkey et al., 2020), suggesting GABA/glutamate could co-release to pyramidal neurons in adult mice. (2) Coexistence of VGLUT2 and VGAT has been found in excitatory mossy fiber terminals of hippocampal CA3 region, cerebellar mossy fiber terminals and GABAergic basket cells terminals in postnatal day 5–15 rat but not adult, indicating co-localization of glutamate and GABA in same vesicles at these terminals (Zander et al., 2010). Immunogold staining also showed localization of α/β subunits of GABA_A_R in mossy fiber synapses (Bergersen et al., 2003). Subsequent functional tests found that activation of mossy fiber boutons attached to CA3 neurons could induce GABAergic inhibitory postsynaptic currents (IPSCs) in two-week-old rats (Beltrán and Gutiérrez, 2012; but also see (Cabezas et al., 2012)). (3) Surprisingly, co-expression of GABA and glutamate in mossy fiber terminals was also discovered in hippocampi from adolescents with epilepsy (Sandler and Smith, 1991). In adult rat epilepsy model, a single pentylenetetrazol (PTZ) treatment elicited a fast inhibitory postsynaptic potential (IPSP) component in mossy fiber of CA3 (Gutierrez, 2000), indicating that glutamate and GABA co-release may occur under pathological condition. (4) Furthermore, Omiya et al. found VGLUT3-positive CCK^+^ cell terminals and α1-containing GABA_A_R at invaginating synapse in basal amygdala of adult mice (Omiya et al., 2015). (5)Additionally, one study reported GABA/glutamate co-release from individual vesicles at entopeduncular nucleus-lateral habenula (EP-LHb) synapses, while the other study provide evidence that GABA and glutamate are stored and released from distinct, non-overlapping pools of synaptic vesicles at the same synapse. A follow-up study implemented a combination of optogenetics, electrophysiology and computational modeling determined that glutamate and GABA are co-packaged in the same synaptic vesicles in EP Sst + neurons projecting to the LHb (Kim et al., 2022).

In terms of GABA-GlyR crosstalk, it is facilitated by the vesicular inhibitory amino acid transporter (VIAAT, also known as VGAT), which could package both glycine and GABA into the same synaptic vesicles (Wojcik et al., 2006). The co-expression of GABA and glycine has been observed in rat spinal cord neurons (Ishibashi et al., 2013), adult mouse retina amacrine neurons (Pérez-León et al., 2022), P10-P23 rat auditory brain stem nucleus (Lu et al., 2008) and adult mouse cerebellum (Milanese et al., 2014), indicating potential crosstalk between GABA and GlyR.

Recent technological advances, such as spatiotemporal omics technology (Xu et al., 2022), have enabled brain-wide mapping of the spatial distribution of neurotransmitter transporters and synthetic enzymes in neurons, providing clearer insights into potential sites for co-releasing transmitters. However, it is important to recognize that the detection of transcripts for neurotransmitter synthetic enzymes does not necessarily imply the presence of the enzymes themselves. Therefore, functional and protein-level verification about neurotransmitter-receptor crosstalk in these neural circuits is warranted.

### 4.2 Non-postsynaptic locations

Neurotransmitter-receptor interaction could also been observed at non-postsynaptic locations, including extrasynaptic and presynaptic sites (Figure 1B). Neurotransmitters released at central synapses may exceed the capacity for removal by reuptake and degradation, diffuse beyond their intended synaptic cleft and activate receptors at adjacent synapses or extrasynaptic sites. The concentration of extrasynaptic neurotransmitter is at low-micromolar level under physiological condition. For instance, the concentration of extrasynaptic glycine and glutamate is around 1–2.5 μM in striatum of conscious rats (Harsing and Matyus, 2013). But under circumstances such as high synaptic activity, or pathological conditions like stroke, epilepsy and transporter dysfunctions, the concentration of extrasynaptic neurotransmitter spillover could reach to submillimolar level. For example, glutamate concentrations can increase to up to 200 μM in the plasma of ischemic stroke patients (Castillo et al., 2016). Glutamate spillover has been found at ectopic sites or induced by high frequency stimulation from parallel fiber synapses in cerebellum and the olfactory bulb of rat (Isaacson, 1999; Balakrishnan et al., 2014). Mutations in the glutamate transporter GLAST (EAAT1) lead to transporter dysfunction and seizures in human (Jen et al., 2005). Genetic knockout of the glutamate transporter GLT-1 (EAAT2) in mice leads to severe epilepsy and increased extracellular glutamate levels (Tanaka et al., 1997).

Ionotropic receptors at extrasynaptic sites exhibit relatively high affinity for neurotransmitter, given the relative low concentration of neurotransmitter spillover. In adult hippocampus and cortex, synaptic NMDAR are primarily composed of the low affinity GluN2A-containing subtype for glutamate and glycine, whereas extrasynaptic NMDAR are predominantly the high affinity GluN2B-containing subtype for both neurotransmitters (Ge and Wang, 2023). Synaptic GABA_A_Rs typically consist of α1-3, β2-3 and γ2 subunits, which exhibit low affinity to GABA and mediate fast phasic currents. In contrast, extrasynaptic GABA_A_Rs often contain α4-6, β2-3 and/or *δ* subunits, enabling them to respond to low ambient GABA and regulate slow tonic currents (Brickley and Mody, 2012; Liu et al., 2018). For instance, extrasynaptic α5-containing GABA_A_Rs have been detected at hippocampal pyramidal cells from P56-P84 mice (Magnin et al., 2019). In cultured hippocampal neurons, the glutamate analog AP5 potentiated both phasic inhibitory postsynaptic currents and tonic currents (Wen et al., 2022), suggesting that allosteric modulation occurs at both synaptic and extrasynaptic GABA_A_R. Also, the α6-containing GABA_A_R could be activated by GABA spillover at cerebellum of 12-day-old rats (Rossi and Hamann, 1998). Similarly, glutamate spillover has been reported in cerebellar climbing fiber to molecular layer interneurons from P12-P25 mice (Malhotra et al., 2021). These results suggest potential modulatory effect of glutamate on extrasynaptic GABA_A_R. Interestingly, GABA released from the histamine/GABA co-releasing neurons in hypothalamic tuberomammillary nucleus (TMN) acts on extrasynaptic GABA_A_R (Yu et al., 2015), though whether histamine could modulate these GABA_A_R needs further investigation. Functional extrasynaptic α2 or α3 homomeric GlyRs have been found in hippocampus from 3-month-old rats (Aroeira et al., 2011), and in dorsal striatum, prefrontal cortex (PFC) and hippocampus from P21–P50 mice (McCracken et al., 2017). But the glycine EC_50_ were reported around 500 μM in these regions, and the mechanisms by which GABA or glutamate spillover modulates extrasynaptic GlyRs remains to be elucidated.

Accumulating studies have demonstrated the presence of ionotropic receptors at presynaptic sites, acting as autoreceptors. Presynaptic GABA_A_Rs have been identified at thalamocortical glutamatergic terminals of P28-P30 rats (Wang et al., 2019). It has also been reported that α2 subunit and *δ* subunit containing GABA_A_R are positioned at hippocampal mossy fiber boutons, where they could co-release GABA and glutamate in 3–12 week-old rats as mentioned before (Ruiz et al., 2003; Alle and Geiger, 2007; Ruiz et al., 2010). These findings suggest that presynaptic GABA_A_R could function as autoreceptors for the co-released neurotransmitters. Similarly, NMDAR composed of GluN2A, GluN2B or GluN3A have also been identified as presynaptic autoreceptor in various regions, including rat cerebellar parallel fiber-Purkinje cell synapses, the spinal cord dorsal horn, hippocampal glutamatergic terminals, and the mice visual cortex at different ages (Liu et al., 1994; Charton et al., 1999; Bidoret et al., 2009; Larsen et al., 2011; Musante et al., 2011). The activation of presynaptic NMDAR is facilitated by the co-release of glycine from glutamatergic synapses (Cubelos et al., 2005; Raiteri et al., 2005; Muller et al., 2013; Cubelos et al., 2014).

Presynaptic homomeric GlyRs have been discovered in rat excitatory synapse, including calyx of Held in MNTB, spinal cords and ventral tegmental area (VTA) GABAergic neurons during development (Colin et al., 1998; Turecek and Trussell, 2001; Ye et al., 2004; Hruskova et al., 2012). The release of glutamate or GABA in these synapses enables the crosstalk with presynaptic GlyR. These findings suggest that presynaptic ionotropic receptors, such as GABA_A_R, NMDAR and GlyR, could be modulated by presynaptic neurotransmitters co-release or spillover.

## 5 Functional implications of neurotransmitter-receptor crosstalk

Among the neurotransmitter-receptor crosstalks listed in Table 1, particularly those involving glycine-NMDAR, glutamate-GABA_A_R, and GABA-GlyR, a common feature is that the cross-matched ligand consistently acts in the same direction as the orthosteric neurotransmitter. For instance, glycine enhances NMDAR function together with glutamate, glutamate potentiates GABA_A_R with GABA, and GABA facilitates GlyR as glycine. This synergistic action observed in the crosstalk between different neurotransmitters may have important physiological and functional implications for neural circuits. E/I balance is the equilibrium between excitatory and inhibitory synaptic inputs within neural circuits. This balance is essential for maintaining optimal neuronal firing rates and overall network stability. Disruption of the E/I balance can contribute to pathogenesis of various neurological disorders characterized by hyperactivity in neural circuits, including epilepsy, ASD and schizophrenia characterized by social deficits.

Activated by the principal excitatory neurotransmitter glutamate, NMDAR is one of the major ionotropic glutamate receptors responsible for excitatory synaptic inputs. Missense mutations in the *GRIN1* gene, which encodes the GluN1 subunit of NMDAR targeting by glycine, have been associated with epileptic encephalopathy or schizophrenia (Ohba et al., 2015; Ju and Cui, 2016). Transgenic *Grin1*^neo-/-^ mice exhibited schizophrenic-like phenotypes, including hyperlocomotor activity, increased stereotypic movement, impaired sensorimotor gating and reduced social interactions (Gandal et al., 2012). The NMDAR hypoactivity mediated GABAergic inhibitory dysfunction was believed associating with pathogenesis of schizophrenia (Cohen et al., 2015). The D481N mutation in GluN1 lead to marked reduction in glycine co-agonist affinity at the NMDAR. Homozygous *Grin1*^D481N^ mice exhibited impairment in hippocampal long-term potentiation (LTP), a synaptic plasticity mechanism underlying learning and memory (Bliss and Collingridge, 1993). Furthermore, these mice showed significant deficits in hippocampus dependent spatial learning task, such as Morris Water Maze, and reduced sensitivity to NMDA-induced seizures (Kew et al., 2000). Importantly, restoring the expression of wild type GluN1 in adult mice with congenital loss-of-function allele of *Grin1* could rescue most of the behavioral phenotypes (Mielnik et al., 2021). Interestingly, a recent study reported that injection of HA-996, a blocker of the glycine-binding site on NMDAR, in periaqueductal gray (PAG) of juvenile rat could interfere the social play behavior, while blocking of astrocytic glycine transporter 1 (GlyT1) inhibitor ALX-5407 could reverse the deficits (Dvorzhak et al., 2024). It should be noted that D-serine or another partial agonist at the glycine-binding site of NMDAR, D-cycloserine, could enhance hippocampal LTP in wild-type rats and alleviate social abnormality in ASD mouse models (Yang et al., 2003; Won et al., 2012; Li et al., 2015). In conclusion, glycine-NMDAR crosstalk is crucial for maintaining E/I balance and facilitating synaptic plasticity, both of which are essential for cognitive processes like spatial learning and social interaction.

GABA_A_R is the main target of treating epilepsy, insomnia, anxiety and ASD characterized by E/I imbalance (Ghit et al., 2021; Vien et al., 2015). As for glutamate-GABA_A_R crosstalk, although there is no available inhibitor specifically blocking the binding of glutamate to GABA_A_R, two lines of transgenic knock-in (KI) mice carrying different mutations that impaired the glutamate allosteric potentiation on the GABA_A_R, have been generated in two recent studies (Wen et al., 2022; Du et al., 2023). Wen et al. generated KI mice carrying a mutation in the β2 subunit of GABA_A_R (β2_E181G_). Du et al. generated KI mice harboring another mutation in the β3 subunit (β3_E182G_). These two mutations effectively disrupted the allosteric potentiation by glutamate without affecting the GABA-mediated activation of GABA_A_R. In β2_E181G_ KI mice, electrophysiological recording in CA1 neurons of hippocampal slice from these mice confirming the impairment of glutamate or glutamate-like AP5 potentiation of GABA_A_R current. Since the GABA_A_R current was induced by either micropressure injection of GABA or electrical theta-burst stimulation, indicating a predominant extrasynaptic GABA_A_R component. The β2_E181G_ KI mice exhibited behavioral phenotypes indicative of increased neuronal excitability, including heightened sensitivity to noxious mechanical and temperature stimuli and a reduced threshold for seizure induction by kainate acid. More comprehensive tests were conducted on β3_E182G_ KI mice. The β3_E182G_ KI mice also had increased susceptibility to seizures induced by kainate acid, and decreased thresholds to both pressure and temperature, indicating elevated E/I imbalance. Furthermore, β3_E182G_ KI mice exhibited enhanced hippocampus-related learning and memory, and impaired social interactions. These mice also showed reduced anxiety-like behaviors. Importantly, re-expression of wild-type α1β3-containing GABA_A_Rs in the dorsal hippocampal CA1 was sufficient to rescue the observed abnormalities in glutamate potentiation and behavioral deficits, supporting the critical role of extrasynaptic glutamate-GABA_A_R crosstalk in maintaining E/I balance and normal brain function.

Glycine-gated GlyR is also an important inhibitory ionotropic receptor in mammalian spinal cord and brain. Mutations in α1 subunit of GlyR have been linked to hyperekplexia characterized by exaggerated startle reflex and increased muscle tone. Mutations in the α2 subunit are associated with ASD (Pilorge et al., 2016; Chen X. et al., 2022). As for GABA-GlyR or glutamate-GlyR crosstalk, since the exact binding sites are still unknown and there are no specific antagonists targeting the binding sites, or transgenic animals with binding site mutation, the physiological role of GABA-GlyR or glutamate-GlyR crosstalk is largely elusive. In rat MNTB where GABA was co-packaging with glycine in same synaptic vesicles, Lu et al. demonstrated that co-releasing GABA could enhance the temporal precision of glycine inhibition, which might be important for sound localization (Lu et al., 2008). Therefore, the physiological significance of GABA or glutamate modulating GlyR requires further investigation.

Under pathological conditions such as ischemia or epilepsy, increased glycine and glutamate concentrations due to spillover can lead to excessive activation of extrasynaptic GluN2B-dependent NMDAR and downstream signaling, potentially contributing to excitotoxicity and neuronal damage (Lasley, 1991; Ge et al., 2020; Ge and Wang, 2023) Pathological spillover can also result from altered expression or function of neurotransmitter transporters, such as the excitatory amino acid transporters (EAATs) for glutamate. Dysfunction of these transporters can lead to increased extracellular levels of glutamate, excessively promoting receptor activation and potentially leading to neurotoxic outcomes. For example, mutations and dysfunction of EAAT2 (GLT-1) have been linked to epilepsy and schizophrenia (Rakhade and Loeb, 2008; Wang et al., 2022). Animal studies demonstrated that EAAT2 dysfunction can lead to increased seizure and cortical injury susceptibility due to impaired glutamate clearance from synapses (Tanaka et al., 1997). However, Nong et al. demonstrated that glycine treatment at 100 μM, but not 1 μM, primes the NMDAR for clathrin-dependent internalization, a process that requires subsequent activation by glutamate (Nong et al., 2003). By regulating the surface expression of NMDARs, glycine-NMDAR crosstalk may help mitigate excitotoxic damage during conditions like stroke or seizure.

As summarized in Table 1, the consequence of neurotransmitter-receptor crosstalk disruption have been always linked to pathological phenotypes, including social deficits and seizures, in transgenic knock-in mice with mutations in cross-matched ligand binding sites. A critical question that arises is how to investigate the potential causal relationship between neurotransmitter-receptor crosstalk and cognitive functions under physiological and psychological conditions during behavior? One direct approach is the development of novel antagonists that specifically target on the cross-matched ligand binding sites of receptor and preventing the potentiation without affecting orthosteric neurotransmitter activation. But such antagonists are often lacking, as their development requires the knowledge of binding sites based on co-crystallization of the ligand and receptor, which is challenging. As mentioned above and summarized in Table 1, another approach involves using transgenic knock-in mice with mutations in the cross-matched ligand binding site of the receptor, leaving the orthosteric neurotransmitter binding unaffected. However, this method has several limitations, including potential developmental compensation by other receptors or signaling pathways, as well as difficulties in manipulating the system temporally and spatially across the whole brain. Given that these neurotransmission crosstalks phenomenon occur in various neural circuits and at different cellular positions across developmental stages, a more targeted approach is necessary. For example, in order to study the role of neurotransmitter-receptor crosstalk in certain neural circuit in regulating social behaviors, one could use transgenic mice with a tamoxifen-inducible system to reduce the VGLUT gene, flanked by loxP sites, by injecting a VGAT-Cre-containing virus into specific neural circuits to inhibit glutamate-GABA_A_R crosstalk in a temporally and spatially controlled manner. Therefore, a combination of these methods is required to fully understand the physiological and pathological role of neurotransmitter-receptor crosstalk. It is crucial to develop potential tools for manipulating neurotransmitter-receptor crosstalk and observe the impact on network dynamics during behavior. Studying of the crosstalk at network level will be an important direction for future research, involving the modeling of specific networks and sub-networks.

## 6 Discussion

The review has discussed the concept of neurotransmitter-receptor crosstalk, focusing on the molecular and cellular mechanisms in both physiological and pathological conditions. The review has also highlighted the importance of the crosstalk in maintaining the E/I balance in the nervous system. Disruption of this balance can contribute to neurological disorders such as epilepsy, ASD and schizophrenia. To further investigate the functional implications of neurotransmitter-receptor crosstalk, the review has discussed the use of transgenic mice and other techniques to study the causal link between crosstalk and cognitive functions, as well as their implications in physiological and psychological conditions.

However, there are still many challenges in studying neurotransmitter-receptor crosstalk. For example, the specific binding sites for many crosstalk interactions are still unknown, and there is a lack of specific tools for manipulating crosstalk *in vivo*. For the therapeutic significance, extension of the current rodent model study to non-human primates or human subject is necessary. Future research is needed to address these challenges and to further elucidate the functional roles of neurotransmitter-receptor crosstalk in health and disease.

To conclude, the study of neurotransmitter-receptor crosstalk has already yielded important insights into the complexity of neural signaling. Understanding how neurotransmitter-receptor crosstalk contributes to E/I balance is critical for developing new therapeutic strategies for social disorders in the future.
